# Editorial: Microbial Stress Responses: Antioxidants, the Plasma Membrane, and Beyond

**DOI:** 10.3389/fmicb.2022.891964

**Published:** 2022-06-02

**Authors:** Sukesh Chander Sharma, Joaquin Arino, Amparo Pascual-Ahuir, Jose M. Mulet, Cristina Mazzoni

**Affiliations:** ^1^Department of Biochemistry, Panjab University, Chandigarh, India; ^2^Department de Bioquimica I Biologia Molecular, Universitat Autonoma de Barcelona, Cerdanyola del Valles, Spain; ^3^Instituto de Biología Molecular y Celular de Plantas, Universitat Politècnica de València-Consejo Superior de Investigaciones Científicas, Valencia, Spain; ^4^Department of Biology and Biotechnologies “C. Darwin”, Sapienza University of Rome, Rome, Italy

**Keywords:** oxidative stress, yeasts, transporter, antioxidant, membrane

Over the past decade, our understanding of the mechanisms involved in microbial responses to stressful environmental conditions have expanded greatly. We are now able to observe both microbial toxicity sensing and microbial adaptations to environmental perturbation. During stressful situations, such as extremes in pH, temperature, humidity and pressure, the microbial cell membrane is always the first target both in bacteria and fungi (Guan and Liu, 2020; Lanze et al., 2020). As the activities of cell membranes are crucial in the functioning of all cells, it is essential to study them during periods of stress, in particular, how the functioning of ATPases is disturbed, whether changes are observed in cation uptake or if membrane potentials are drastically altered. Emerging technologies, such as transcriptomics, proteomics, metabolomics, lipidomics and glycomics, can help us to understand the molecular mechanisms that interplay within the cell membrane during microbial exposure to stressful conditions. Investigation into aspects of the cell membrane, such as membrane raft structure characterization, ion homeostasis, and the role of ion transporters and antioxidants could shed valuable light on the holistic microbial response to stress. Frontiers in Microbiology took initiative in bringing out a special issue on this cutting-edge theme. In this Research Topic we collected 1 review and 9 original research articles which combined give an overview on the current status of this field.

In the review (Ferraz et al.), the authors consider the harsh conditions applied to microorganisms during industrial fermentations and how the plasma membrane is involved in strains productivity. They present an overview of membrane engineering strategies applied to *Saccharomyces cerevisiae* to enhance its fitness under industrially relevant conditions as well as strategies to increase microbial production of the metabolites of interest. The knowledge on this topic is not yet exhaustive and will require the collaboration of different disciplines ranging from lipidomics, molecular dynamics simulations and membrane biophysics to gain more information on the plasma membrane that could be used at systems level.

The study of Camponenschi et al. deals with the effects of light on oxidative stress and lipid biosynthesis in the *Kluyveromyces lactis* yeast. Although yeast lacks specialized light-sensing proteins, it has been reported that budding yeast *S. cerevisiae* respond to light by increasing hydrogen peroxide level and triggering nuclear translocation of the stress-response transcription factor Msn2 (Bodvard et al., 2011). In this paper, the authors show a role in light response of the regulatory factor KlMga2, involved in response to ROS, life span, and general cellular fitness (Santomartino et al., 2019), opening new perspectives on the role of lipids and membranes in the response to light stress.

Membranes posses a unique feature called “homeoviscous adaptation” in order to maintain normal function of that system while undergoing stressful conditions. The key need for this feature is dependent upon the compositional changes of membrane matrix to control its fluidity. Paullucci et al. studied the long term response to temperature stress in Synorhizobium in Medicago sativa is due *de novo* synthesis of fatty acid composition of phospholipids and where fluidity played a key role in symbiosis.

Da Silva Rovida et al. addressed the problem of the toxicity of herbicides, used in agriculture to control weeds, toward non-target organisms. Some herbicides have electronegative and oxidizing chemical elements, such as fluorine and chlorine that can affect the stress response genes in microorganisms compromising their physical structure and causing oxidative stress through the generation of reactive oxygen species (ROS) which interact with the cell membrane and can cause lipid peroxidation. As a way of preventing or reducing these types of imbalances, bacteria have developed response systems to protect membrane integrity, such as modulating the activity of antioxidant enzymes. To test the toxicity of various commercial herbicides, the authors studied the modulation of antioxidant mechanisms of a *Pseudomonas* strain collected from biofilms in a specific herbicide packaging washing tank. This strain shows a higher tolerance to herbicides and biofilms containing this strain could protect the herbicide-sensitive isolates, opening a possibility of using biofilms containing such a kind of resistant bacterial strains for the bioremediation of contaminated areas.

For Gram-negative bacteria, the cell envelope structure, composed by the outer membrane (OM), the peptidoglycan and the inner membrane (IM), has a multitude of functions and it's fundamental to maintain the envelope homeostasis through a sophisticated regulatory network that comprises Cpx two-component regulatory system (Hews et al., 2019). Tsviklist et al. found that *E. coli* cells require the integrity of the two-component system Cpx envelope stress response especially during respiration. Moreover, the authors demonstrate that the Cpx two-component system serves as a sentry of inner membrane protein biogenesis, ensuring the function of large envelope protein complexes and maintaining the cellular energy status of the cell.

Some papers focused on stress induced by environmental factors such as temperature, salt or water stress. Goh et al. provide a molecular description of the stress response in *E. coli*. The paper describes how cold shock induces translational stress response via GTPase Bip A, a factor affecting the growth, swimming mobility and ribosome assembly. These results suggest that BipA is a *bona fide* 50s assembly factor in the bacterial ribosome biogenesis. Liu et al. investigated the effect of salt and osmotic stress on the phytofungus *Setosphaeria turcica*, a plant pathogen which causes the corn leaf blight disease. The report shows that osmotic stress increases the fungal pathogeneicity, and that this is dependent on the HOG-MAPK pathway. Moreover, the aquaglyceroporin StFPS1, a target of regulation of the HOG-MAPK pathway, is involved in the penetration ability of *S. turcica*. Following a similar approach (the interplay between environmental stress and pathogenicity). Li F. et al. investigated this phenomenon in the *Escherichia coli* BW25113 bacteria pointing to a pivotal role of trehalose in this process, This is of major interest as trehalose has also been found to have an important role in salt stress in fungi (Mulet et al., 2004). Li P.-S. et al. brought new insight into the rhizosphere bacterium *Rahnella aquatilis* JZ-GX1 and found that is moderately halophilic, and that the secretion of exopolysaccharides has a role in salt tolerance. *R. aquatilis* is a good growth promoter for plants, providing aknowledge that may be useful for agronomy. Finally Luo and Tian contributed with a report comparing two different strains of *Epichloë sinensis* aiming at gaining knowledge on the environmental adaptation of this endophyte and the role of phytohormones.

The most of the article are with the theme of the issue. The role of another important antioxidant molecular GSH has not found its place. Role of membrane rafts, alterations in the lipid- protein interactions, role of sterols, signaling lipids, their dynamics, homeostasis, lipidome, glycome, and transbilayer asymmetric distribution. Aforementioned studies can further help to gain more understanding of the role of plasma membrane, antioxidants in stress tolerance.

## Author Contributions

SS, JM, and CM wrote the draft of the manuscript. SS, JA, AP-A, JM, and CM contributed to manuscript revision. All authors contributed to the article and approved the submitted version.

## Conflict of Interest

The authors declare that the research was conducted in the absence of any commercial or financial relationships that could be construed as a potential conflict of interest.

## Publisher's Note

All claims expressed in this article are solely those of the authors and do not necessarily represent those of their affiliated organizations, or those of the publisher, the editors and the reviewers. Any product that may be evaluated in this article, or claim that may be made by its manufacturer, is not guaranteed or endorsed by the publisher.
